# Identification of Recurrence-Related mRNAs and Noncoding RNAs in Hepatocellular Carcinoma Following Liver Transplantation

**DOI:** 10.5152/tjg.2023.22656

**Published:** 2023-04-01

**Authors:** Qiang Zeng, Jinglin Cao, Yishan Niu, Xin Zhao, Yang Wang, Wenpeng Liu, Baowang Liu, Yajie Chen, Yize Fan, Jian Dou

**Affiliations:** 1 Department of Hepatobiliary Surgery, Third Hospital of Hebei Medical University, Shijiazhuang, China

**Keywords:** Hepatocellular carcinoma, liver transplantation, recurrence, lncRNA, circRNA

## Abstract

**Background:**

This study aimed to determine whether altered mRNA, long non-coding RNA, and circular RNA expression is related to hepatocellular carcinoma recurrence after liver transplantation.

**Methods:**

Five recurrent and 5 non-recurrent primary hepatocellular carcinoma samples were used to perform RNA sequencing. Then, differentially expressed mRNAs, differentially expressed long non-coding RNAs, and differentially expressed circular RNAs between recurrent group and non-recurrent group were identified. In addition, differentially expressed long non-coding RNA/differentially expressed circular RNA–differentially expressed mRNA co-expression network, and competing endogenous RNA (differentially expressed circular RNA/differentially expressed long non-coding RNA–miRNA–differentially expressed mRNA) regulatory network were constructed. Finally, real-time quantitative polymerase chain reaction was performed for verification.

**Results:**

Five hundred forty-one differentially expressed mRNAs, 239 differentially expressed long non-coding RNAs, and 16 differentially expressed circular RNAs in the recurrent group were obtained. Gene set enrichment analysis indicated that these differentially expressed mRNAs may affect hepatocellular carcinoma recurrence through multiple pathways, such as E2F, epithelial–mesenchymal transition, G2M, and oxidative phosphorylation. Then, 993 differentially expressed long non-coding RNA–differentially expressed mRNA co-expression pairs and 28 differentially expressed circular RNA/differentially expressed mRNA co-expression pairs were obtained. The competing endogenous RNA network contained 10 circular RNA–miRNA pairs, 12 long non-coding RNA–miRNA pairs, and 36 miRNA–mRNA pairs. Real-time quantitative polymerase chain reaction results showed the same expression trend as RNA-seq results.

**Conclusion:**

Our results reveal key mRNAs, long non-coding RNAs, and circular RNAs associated with recurrence in hepatocellular carcinoma patients after liver transplantation and lay the foundation for understanding the molecular mechanism of hepatocellular carcinoma recurrence after liver transplantation.

**Main Points**

- Our results reveal key mRNAs, long non-coding RNAs, and circular RNAs associated with recurrence in hepatocellular carcinoma (HCC) patients after liver transplantation.
- Differentially expressed mRNAs may affect HCC recurrence through multiple pathways.
- This is helpful for postoperative recurrence monitoring of HCC.

## Introduction

Surgical resection and liver transplantation (LT) are common treatments for hepatocellular carcinoma (HCC). Hepatocellular carcinoma has a higher recurrence rate. However, the precise mechanisms underlying the recurrence of HCC are still unknown. Therefore, identifying reliable and accurate predictive markers to screen out which subset of patients with HCC is vulnerable to develop recurrence is urgently needed.

Studies have found that long non-coding RNAs (lncRNAs) and circular RNAs (circRNAs) are involved in the development of HCC. MALAT1 could enhance hepatocarcinoma cell growth.^1^ The cSMARCA5 inhibits the growth and migration of HCC cells and is a potential therapeutic target.^2^ The circAKT3 is associated with HCC recurrence and mortality.^3^ In the present study, we analyzed the expression of lncRNAs, circRNAs, and mRNAs in HCC recurrence after LT to obtain the differentially expressed (DE) mRNAs, DElncRNAs, and DEcircRNAs, aiming to identify early-phase biomarkers to predict HCC recurrence after LT. In addition, DElncRNA/DEcircRNA–DEmRNA co-expression network and competing endogenous RNA (ceRNA, DEcircRNA/DElncRNA–miRNA–DEmRNA) regulatory network were constructed.

Through this work, we hope to provide some insights into the personalized treatment of HCC and improve the strategy of postoperative recurrence monitoring in HCC patients.

## Materials and Methods

### Subjects and Samples

Five recurrent and 5 non-recurrent primary HCC samples obtained from 10 patients who underwent LT were included in the study. All samples were collected after obtaining written informed consent from every participant. This study was approved by the ethics committee of the Third Hospital of Hebei Medical University and performed in accordance with the Declaration of Helsinki. Peripheral whole blood (2.5 mL) drawn from each subject was used for RNA extraction.

### RNA Sequencing, Identification of Differentially Expressed mRNAs, Differentially Expressed Long Non-coding RNAs, and Differentially Expressed Circular RNAs

With PAXgene® RNA blood tubes, total RNA was extracted from samples. Based on the DNBSEQ platform (PE100 strategy), sequencing was performed. The Fastp was used to trim 5ʹ and 3ʹ segments of reads to remove bases with mass <20 and delete reads with N >10%. HISAT2 was applied to align the clean reads with the human reference genome (GRCh38). The expression of mRNAs and lncRNAs was normalized and outputted with StringTie. Then, the CIRIquant software was used to predict circRNAs. DEseq2 was used to identify DEmRNAs, DElncRNAs, and DEcircRNAs in recurrent HCC (reHCC) with |log_2_FoldChange| (|log_2_FC|) >1 and *P* < .05.

### Functional Analysis

GeneCodis4.0 (https://genecodis.genyo.es/) was used to perform Gene Ontology (GO) and Kyoto Encyclopedia of Genes and Genomes (KEGG) analysis for DEmRNAs (*P*_adj. < .05). Gene set enrichment analysis (GSEA) was conducted by using GSEA (version 4.1.0) to find out enriched gene sets (*P*-value < .05). Hallmark gene sets (h.all.v7.3.symbols.gmt) were downloaded from MSigDB.

### Differentially Expressed Long Non-coding RNA/Differentially Expressed Circular RNA–Differentially Expressed mRNA Network

Differentially expressed long non-coding RNA/DEcircRNA*–*DEmRNA pairs with |*r*| ≥ 0.9 and *P* < .001 were defined as co-expressed DElncRNA/DEcircRNA*–*DEmRNA pairs. Differentially expressed long non-coding RNA/DEcircRNA*–*DEmRNA networks were visualized by using Cytoscape. Gene Ontology and KEGG enrichment analyses of the DEmRNAs co-expressed with DElncRNA were performed with GeneCodis4.0 (*P*_adj. < .05).

### Differentially Expressed Circular RNA/Differentially Expressed Long Non-coding RNA–miRNA–Differentially Expressed mRNA (Competing Endogenous RNA) Network

The miRWalk was used to predict the target DEmRNAs of miRNAs. The NPInter v4.0 was used to predict the DElncRNA*–*miRNA interaction pairs. The DEcircRNA*–*miRNA interaction pairs were predicted with targetscan. Then, circRNA/lncRNA*–*miRNA pairs were combined with miRNA*–*mRNA pairs to construct the ceRNA regulatory network.

### Real-time Quantitative Polymerase Chain Reaction

Four recurrent and 6 non-recurrent primary HCC samples obtained from 10 patients who underwent LT were enrolled in this study. Following the manufacturer’s protocol, total RNA was isolated from blood samples with the RNAliquid reagent. The real-time quantitative polymerase chain reaction (RT-qPCR) was performed using SuperReal PreMix Plus (SYBR-Green; Tiangen, Beijing, China). Glyceraldehyde-3-phosphate dehydrogenase (GAPDH) and actin beta (ACTB) were used as endogenous controls. Relative gene expression was analyzed by the 2^–ΔΔCT^ method.

### Statistical Analysis

In this study, DEseq2 (http://www.bioconductor.org/packages/release/bioc/html/DESeq2.html) was used to identify DEmRNAs, DElncRNAs, and DEcircRNAs in reHCC with |log_2_FC| > 1 and *P* < .05. GeneCodis 4.0 (https://genecodis.genyo.es/) was used to perform GO and KEGG analysis for DEmRNAs (*P*_adj < .05). In RT-qPCR, the relative gene expression was analyzed by 2^–ΔΔCT^ method.

## Results

### Differential Expression Analysis

**Figure 1.**
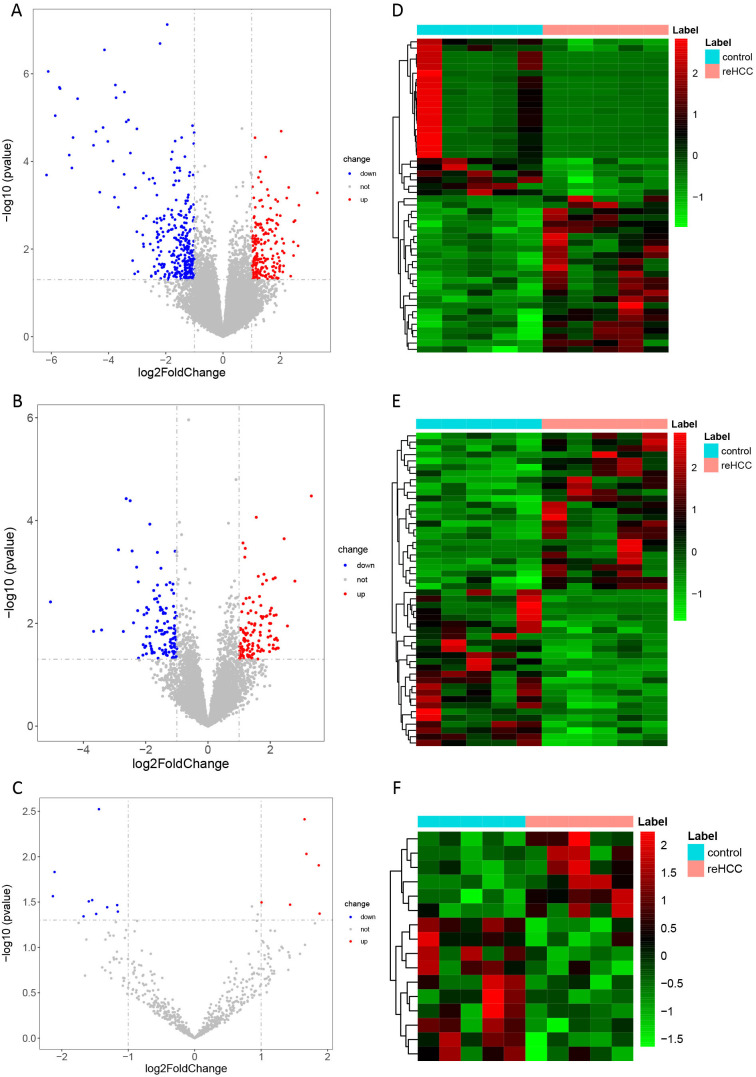
Volcano plot (A–C) and heatmap (D–F) displaying dysregulated mRNAs (A, D), lncRNAs (B, E), and circRNAs (C, F) in HCC recurrence after LT. Rows and columns represent DEmRNAs/DElncRNAs/DEcircRNAs and samples, respectively. The color scale represents the expression levels. circRNAs, circular RNAs; DE, differentially expressed; HCC, hepatocellular carcinoma; lncRNAs, long non-coding RNAs; LT, liver transplantation.

Compared to the non-recurrent group, 541 DEmRNAs, 239 DElncRNAs, and 16 DEcircRNAs in the recurrent group were obtained. The top 10 up- and downregulated DEmRNAs and DElncRNAs and all DEcircRNAs are summarized in Tables 1, 2, and 3, respectively. The volcano plot and heatmap of the top 100 10 up- and downregulated DEmRNAs and DElncRNAs and all DEcircRNAs are shown in Figure 1.

### Functional Analysis of Differentially Expressed mRNAs

**Figure 2.**
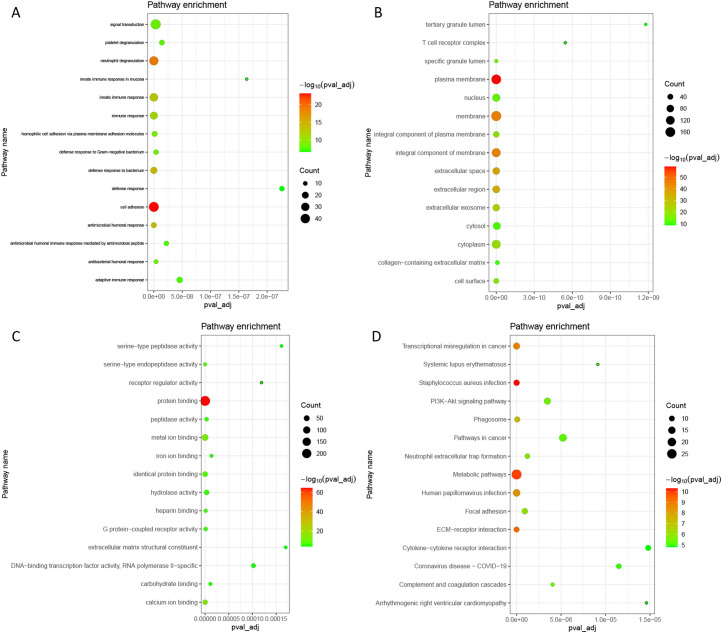
Significantly enriched GO terms and KEGG pathways of DEmRNAs. (A) BP, biological process; (B) CC, cellular component; (C) MF, molecular function; and (D) KEGG pathways. DE, differentially expressed.

Gene Ontology analysis indicated that these DEmRNAs were significantly enriched in cell adhesion (*P* = 3.84E-27), neutrophil degranulation (*P* = 1.47E-22), plasma membrane (*P* = 1.96E-62), protein binding (*P* = 5.00E-68), and calcium ion binding (*P* = 1.90E-17) (Figure 2A, B and C). In the KEGG analysis, several pathways were significantly enriched, including *Staphylococcus aureus* infection (*P* = 2.16E-13), metabolic pathways (*P* = 1.17E-12), and extracellular matrix (ECM)–receptor interaction (*P* = 7.61E-12) (Figure 2D).

### Gene Set Enrichment Analysis

**Figure 3.**
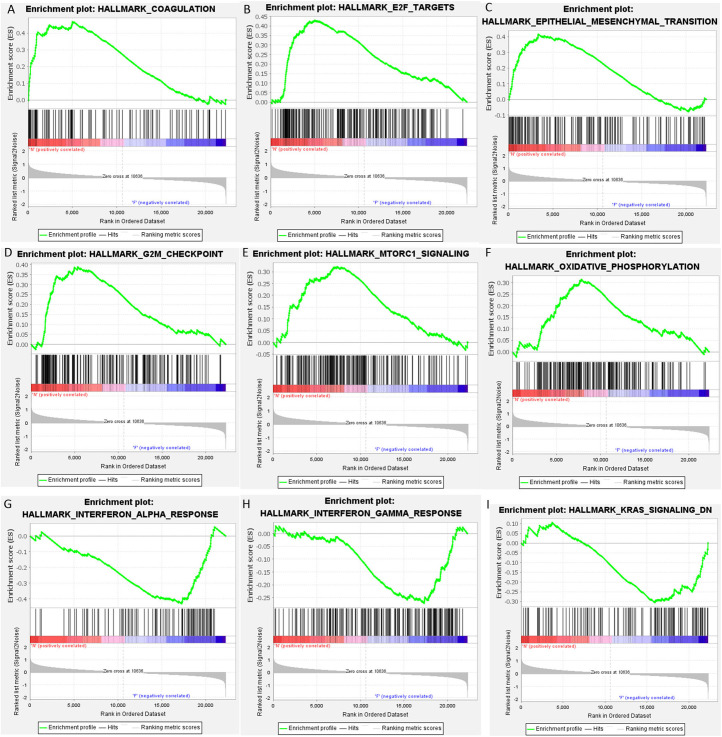
Gene set enrichment analysis illustrated upregulated gene sets in non-recurrent (A–F) and recurrent groups (G–I).

Gene set enrichment analysis showed that 13 gene sets were significantly upregulated in the non-recurrent group, while 5 gene sets were significantly upregulated in the recurrent group (*P* < .05). Among them, the enrichment of gene sets of “COAGULATION,” “E2F TARGETS,” “EPITHELIAL–MESENCHYMAL TRANSITION,” “G2M CHECKPOINT,” “MTORC1 SIGNALING,” “OXIDATIVE PHOSPHORYLATION,” “INTERFERON ALPHA RESPONSE,” “INTERFERON GAMMA RESPONSE,” and “KRAS SIGNALING DN” showed higher significance (*P* < .01) (Figure 3).

### Differentially Expressed Long Non-coding RNA/Expressed Circular RNA–Differentially Expressed mRNA Network and Competing Endogenous RNA Network

**Figure 4.**
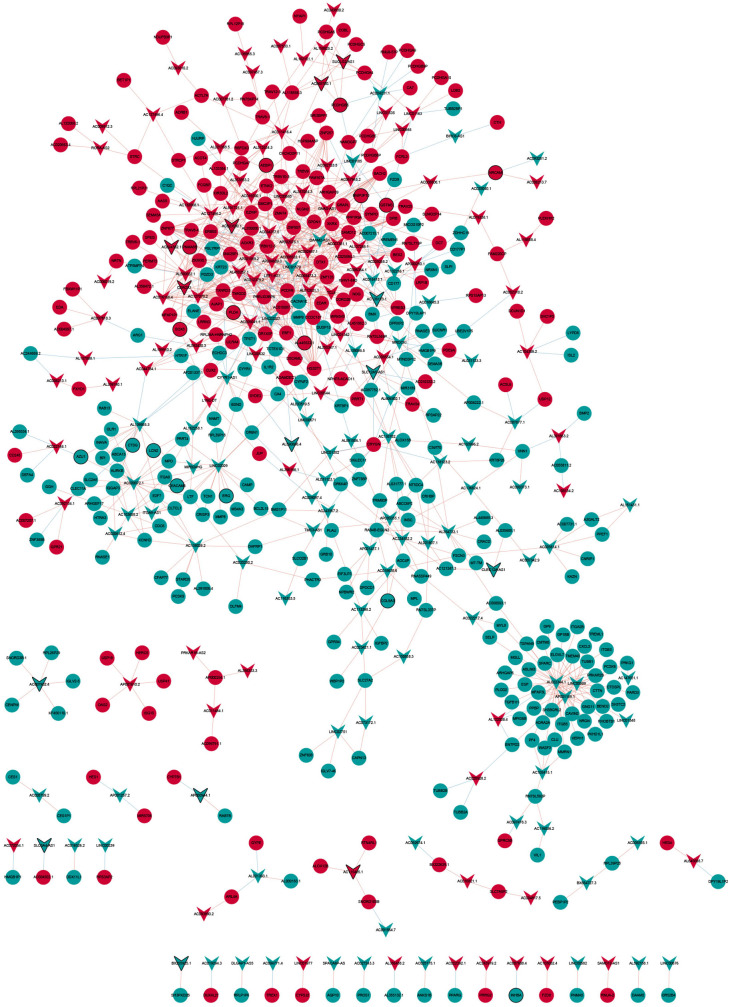
DElncRNA–DEmRNA co-expression network. The inverted triangles and ellipses represent DElncRNAs and DEmRNAs between recurrent group and non-recurrent group, respectively. Red and green colors represent up- and downregulation, respectively. Nodes with black border were DEmRNAs/DElncRNAs derived from top 10 up- and downregulated DEmRNAs/DElncRNAs. DE, differentially expressed; lncRNAs, long non-coding RNAs.

**Figure 5.**
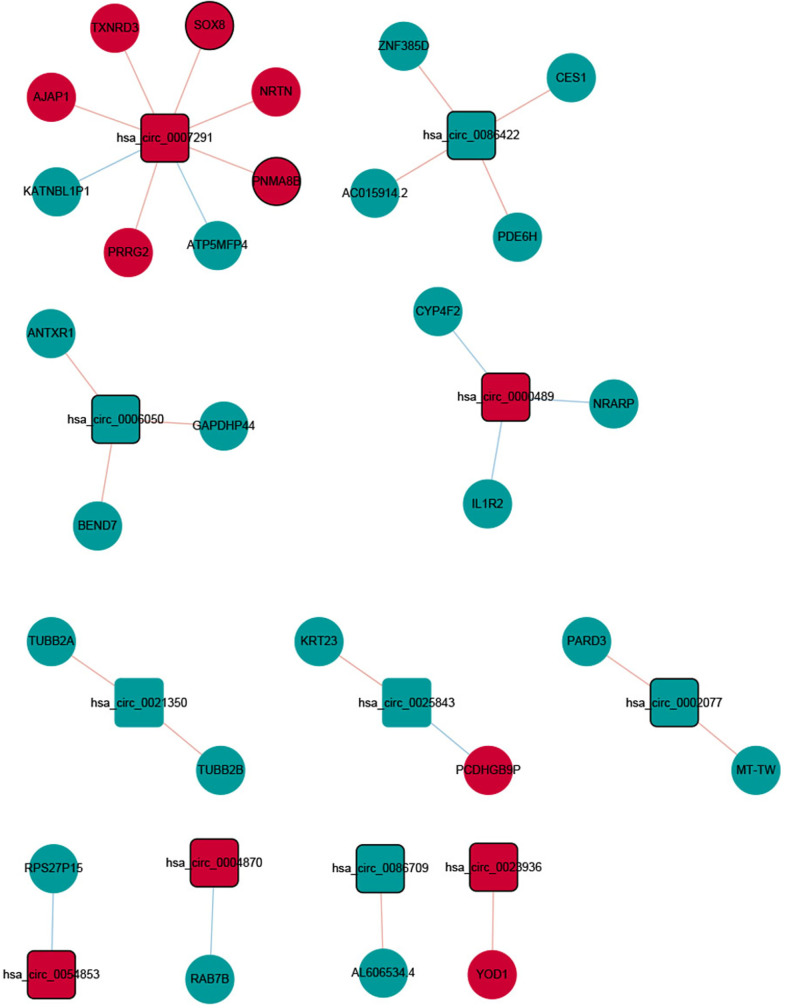
DEcircRNA–DEmRNA co-expression network. The rectangles and ellipses represent DEcircRNAs and DEmRNAs between recurrent group and non-recurrent group, respectively. Red and green color represent up- and downregulation, respectively. Nodes with black border were DEmRNAs/DEcircRNAs derived from top 10 up- and downregulated DEmRNAs/DElncRNAs. DEcircRNA, differentially expressed circular RNAs; DElncRNAs, differentially expressed long non-coding RNAs.

**Figure 6.**
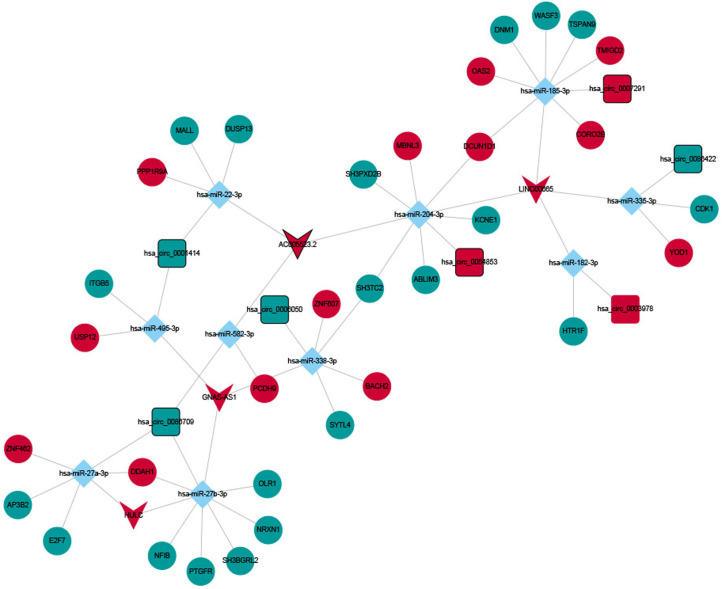
ceRNA (DEcircRNA/DElncRNA–miRNA–DEmRNA) regulatory network. The rectangles, inverted triangles, rhombuses, and ellipses indicate DEcircRNAs, DElncRNAs, miRNAs, and DEmRNAs, respectively. Red and blue colors represent upregulation and downregulation, respectively. Nodes with black border were DEcircRNAs/DElncRNAs/DEmRNAs derived from top 10 up- and downregulated DEcircRNAs/DElncRNAs/DEmRNAs. ceRNA, competing endogenous RNA; DEcircRNA, differentially expressed circular RNAs; DElncRNAs, differentially expressed long non-coding RNAs.

**Supplementary Figure 1.**
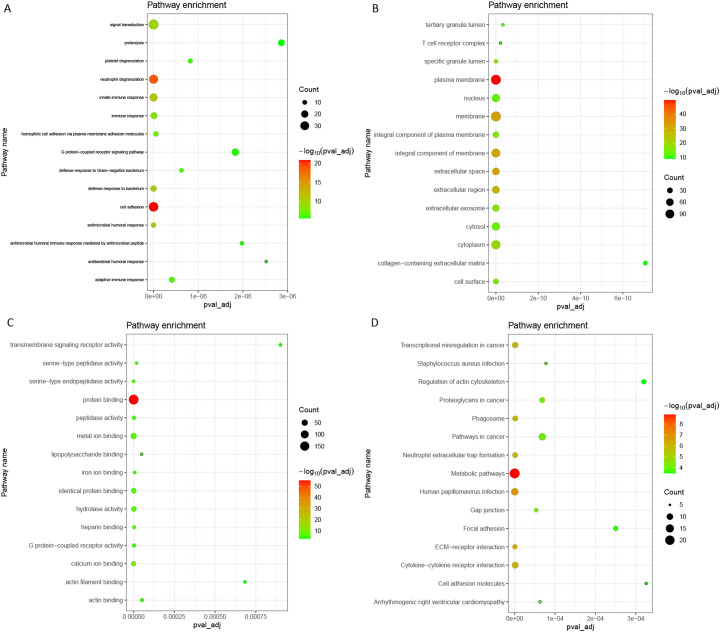
Significantly enriched GO terms and KEGG pathways of DEmRNAs co-expressed with DElncRNAs. (A) BP, biological process; (B) CC, cellular component; (C) MF, molecular function; (D) KEGG pathways.

Nine hundred ninety-three DElncRNA–DEmRNA co-expression pairs including 187 DElncRNAs and 379 DEmRNAs were obtained with |*r*| ≥ 0.9 and *P* < .001 (Figure 4). Among them, AL022344.1 (degree = 41), AC127070.2 (degree = 27), AC092667.1 (degree = 24), 1AC007381.1 (degree = 22), and AC000072.1 (degree = 21) were 5 hub lncRNAs. Gene Ontology analysis indicated that the DEmRNAs co-expressed with DElncRNAs were significantly enriched in cell adhesion (*P* = 9.22E-25), neutrophil degranulation (*P* = 2.92E-22), plasma membrane (*P* = 9.85E-53), protein binding (*P* = 9.18E-59), and calcium ion binding (*P* = 3.82E-14) (Supplementary Figure 1A, 1B, and 1C). In the KEGG analysis, several pathways were significantly enriched, including metabolic pathways (*P* = 6.68E-12), human papillomavirus infection (*P* = 1.71E-09), and ECM–receptor interaction (*P* = 9.85E-09) (Supplementary Figure 1D). In total, 28 DEcircRNA/DEmRNA co-expression pairs including 11 DEcircRNAs and 28 DEmRNAs were obtained with |*r*| ≥ 0.9 and *P* < .001 (Figure 5). The ceRNA network contained 10 circRNA–miRNA pairs, 12 lncRNA–miRNA pairs, and 36 miRNA–mRNA pairs, including 7 circRNAs, 4 lncRNAs, 10 miRNAs, and 33 mRNAs (Figure 6).

### Real-Time Quantitative Polymerase Chain Reaction Validation

**Figure 7.**
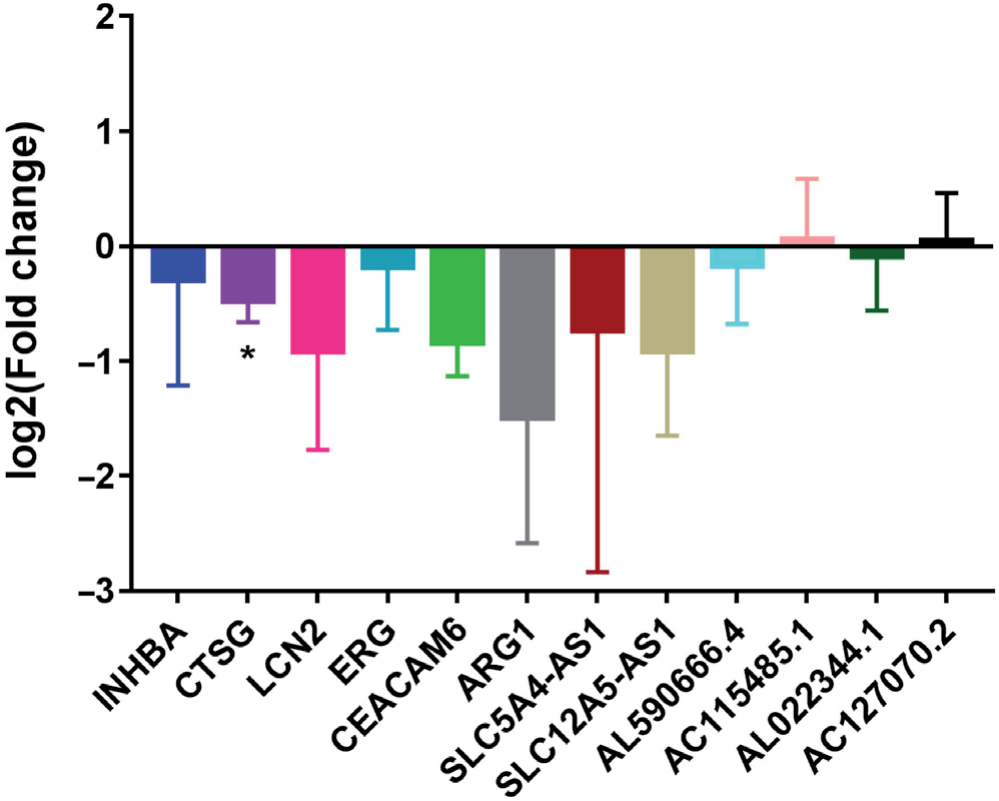
RT-qPCR results of DEmRNAs and DElncRNAs. DElncRNAs, differentially expressed long non-coding RNAs; RT-qPCR, real time quantitative polymerase chain reaction.

Six mRNAs (INHBA, CTSG, LCN2, ERG, CEACAM6, and ARG1) and 6 lncRNAs (SLC5A4-AS1, SLC12A5-AS1, AL590666.4, AC115485.1, AL022344.1, and AC127070.2) were selected randomly to use for RT-qPCR validation. Based on our RNA-seq results, AC115485.1 and AC127070.2 were upregulated, while INHBA, CTSG, LCN2, ERG, CEACAM6, ARG1, SLC5A4-AS1, SLC12A5-AS1, AL590666.4, and AL022344.1 were downregulated in reHCC. It is noted that the RT-qPCR results exhibited the same pattern as our RNA-seq results (Figure 7).

## Discussion

Liver transplantation remains one of the most curative treatment options for HCC, with increasing mortality and morbidity of HCC. Hepatocellular carcinoma recurrence is the main underlying cause for the poor prognosis of HCC following LT. Herein, we identified the genes lncRNAs and circRNAs that are related to tumor recurrence in HCC after LT.

Du et al^4^ indicated that CEACAM6 promoted cisplatin resistance in lung adenocarcinoma and was regulated by microRNA. Roy et al^5^ suggested that CEACAM6 was a biomarker for gastric cancer. Huang et al^6^ indicated that CTSG was a potential marker in oral squamous cell carcinoma. Zou et al^7^ reported that CTSG was associated with overall survival in patients with oral squamous cell carcinoma. Both CEACAM6 and CTSG were downregulated in this study, which indicated the importance of CEACAM6 and CTSG in tumor recurrence in HCC patients after LT. Wu indicated that INHBA plays a role in head and neck squamous cell carcinoma.^8^ Seeruttun et al^9^ showed that INHBA was the most optimally reliable biomarker for diagnosing gastric cancer and lymph node metastasis. Zhang et al^10^ showed that LCN2 was a potential biomarker for radioresistance and recurrence in nasopharyngeal carcinoma. INHBA and LCN2 were downregulated in this study, which implied that INHBA and LCN2 may play crucial roles in tumor recurrence in HCC patients after LT.

Wang et al^11^ indicated that AC068700.1 was upregulated in pheochromocytoma and paraganglioma. Zhang et al^12^ reported that AC005523.2 was downregulated in melanoma and associated with melanoma. Long non-coding RNA IDI2-AS1 was identified to be associated with the progression of colon cancer.^13^ Teng et al^14^ suggested that IDI2-AS1 was related to bone metastasis in breast cancer. These lncRNAs mentioned above were dysregulated in this analysis, indicating they may be involved in HCC recurrence after LT.

Genomic imprinting is one epigenetic phenomenon which is associated with many human diseases or syndromes as well as with various types of cancers. In individuals with a paternally imprinted gene, only the allele inherited from the mother is expressed and vice versa. The PPP1R9A gene is located in a cluster of imprinted genes on human chromosome 7q21, which was upregulated in hepatosplenic T-cell lymphoma.^15^ Downregulation of PPP1R9A was correlated with more advanced cancer stages in squamous cell carcinoma of the head and neck.^16^ The reduced expression of miR-22-3p in breast cancer was associated with tumor size, tumor node metastasis (TNM) stage, and lymph node metastasis, which suppressed breast cancer (BC) cell tumorigenesis.^17^ Xiao et al^18^ suggested that miR-22-3p promotes bladder cancer chemoresistance, which may serve as a new prognostic biomarker for bladder cancer patients. In addition, miR-22-3p is reported to play an essential role in the regulation of HCC progression.^19^ In the ceRNA network, PPP1R9A was a target of hsa-miR-22-3p, and AC005523.2 may act as the sponge of hsa-miR-22-3p to capture PPP1R9A in HCC recurrence after LT.

DCUN1D1 is a transcription factor, which is increased significantly and associated with progression and prognosis of prostate cancer.^20^ MBNL3 was identified to regulate cell invasion of pancreatic ductal adenocarcinoma.^21^ In addition, MBNL3 was reported to promote tumorigenesis and indicate poor prognosis of HCC patients.^22^ Huang et al^23^ reported that miR-204-3p was involved in colorectal cancer metastasis. Decreased miR-204-3p was detected in gastric cancer specimens when compared with non-tumor specimens.^24^ Cui et al^25^ revealed that miR-204-3p inhibits the growth of HCC tumor endothelial cells. In the ceRNA network, MBNL3 and DCUN1D1 were targets of hsa-miR-204-3p, and AC005523.2 and hsa_circ_0054853 may act as the sponges of hsa-miR-204-3p to capture MBNL3 and DCUN1D1 in HCC recurrence after LT.

The sex-determining region Y-box (SOX) family is an important group of transcription factors involved in tumorigenesis and cancer, which critically control cell fate and differentiation in cancer. SOX8 is a member of the SoxE group in the SOX family, which has been reported to function as an oncogene and involved in the progression of triple-negative breast cancer.^26^ High expression of SOX8 was usually associated with a poor prognosis in colorectal cancer.^27^ SOX8 was also overexpressed in chemoresistant patients with tongue squamous cell carcinoma and was associated with poor prognosis.^28^ In addition, highly expressed SOX8 was detected in HCC, which promoted cellular proliferation and enhanced tumor growth in HCC.^29^ Elevated hsa_circ_0007291 was detected in thymoma, which was associated with pathological immune disorder in thymoma.^30^ In this study, hsa_circ_0007291, an upregulated DEcircRNAs that covered the most DEmRNAs, was co-expressed with SOX8, which may indicate that hsa_circ_0007291 was involved in HCC recurrence after LT by regulating SOX8.

However, there are still some limitations in this study. First, the sample size of sequencing is small, and a large number of samples need to be collected for subsequent verification. Second, the specific mechanism of the identified mRNA-, lncRNA-, and circRNA-associated recurrence in HCC patients after LT is still unclear, and a large number of experiments are needed to verify it.

## Conclusions

In conclusion, this study investigated the lncRNA, circRNA, and mRNA expression profiles of tumor recurrence and without tumor recurrence in HCC patients and found altered lncRNA, circRNA, and mRNA expression pattern during the recurrence HCC after LT. Our results reveal key genes circRNAs and lncRNAs associated with recurrence in HCC patients after LT and lay the foundation for understanding the molecular basis of recurrence in HCC patients after LT. The precise mechanisms of the potential biomarkers in recurrence HCC after LT need to be confirmed by further validation or experiments.

## Figures and Tables

**Table 1.** Top 10 Up- and down Regulated DEmRNAs in reHCC

Symbol	log_2_FoldChange	*P*	Regulation
COL9A3	–1.95032	7.52E-08	Down
RBPMS2	–2.20097	2.04E-07	Down
INHBA	–4.14265	2.85E-07	Down
CTSG	–6.10909	8.85E-07	Down
LCN2	–3.76175	1.80E-06	Down
DEFA1B	–5.71755	2.04E-06	Down
DEFA1	–5.6937	2.18E-06	Down
ERG	–3.44512	2.61E-06	Down
CEACAM6	–3.7381	3.52E-06	Down
AZU1	–5.08192	3.70E-06	Down
NRCAM	2.032138	2.04E-05	Up
BNIP3P10	1.119146	2.88E-05	Up
PCDHGB6	1.494117	7.97E-05	Up
GSTM3	1.2965	0.000170194	Up
SOX8	1.238753	0.000232401	Up
AEBP1	1.335882	0.000295183	Up
PLD4	1.032001	0.000379577	Up
AL445523.1	2.295053	0.000392675	Up
PNMA8B	1.549331	0.000437694	Up
MKRN4P	3.294219	0.000521909	Up

DE, differentially expressed; HCC, hepatocellular carcinoma.

**Table 2.** Top 10 Up- and Down Regulated DElncRNAs in reHCC

Symbol	log_2_FoldChange	*P*	Regulation
SLC5A4-AS1	–2.62628	3.75E-05	Down
SLC12A5-AS1	–2.498	4.12E-05	Down
AC022133.2	–1.86644	0.000118	Down
AP000944.1	–2.87486	0.000372	Down
AL590666.4	–2.4357	0.000391	Down
CLEC12A-AS1	–1.05459	0.000395	Down
IDI2-AS1	–1.6241	0.000417	Down
BX255925.1	–2.29065	0.000808	Down
AC138123.1	–1.50979	0.000849	Down
AC007952.4	–2.23922	0.001562	Down
AC010733.1	3.319991	3.33E-05	Up
AC068700.1	1.551749	8.65E-05	Up
AC115485.1	2.446512	0.000226	Up
CEROX1	1.12451	0.000273	Up
AC024588.1	1.198138	0.000348	Up
SUCLG2-AS1	1.183271	0.000515	Up
AC024382.1	1.798147	0.001111	Up
AC023590.1	1.616171	0.001218	Up
AC246817.2	2.160635	0.001299	Up
AC005523.2	2.093487	0.001366	Up

DE, differentially expressed; HCC, hepatocellular carcinoma.

**Table 3. t3-tjg-34-4-394:** All DEcircRNAs in reHCC

Symbol	log_2_FoldChange	*P*	Regulation
hsa_circ_0007291	1.653333	.003875	Up
hsa_circ_0000489	1.681826	.009328	Up
hsa_circ_0004870	1.867304	.012459	Up
hsa_circ_0023936	1.005867	.031915	Up
hsa_circ_0054853	1.435914	.033813	Up
hsa_circ_0003978	1.879863	.042455	Up
hsa_circ_0001414	–1.44067	.002998	Down
hsa_circ_0006050	–2.10853	.014758	Down
hsa_circ_0086422	–2.13363	.02727	Down
hsa_circ_0014624	–1.54235	.030122	Down
hsa_circ_0086709	–1.59348	.031106	Down
hsa_circ_0002077	–1.16601	.034196	Down
hsa_circ_0025843	–1.316 24	.036102	Down
hsa_circ_0021350	–1.15589	.040342	Down
hsa_circ_0000128	–1.48203	.042768	Down
hsa_circ_0039857	–1.6724	.045484	Down

DE, differentially expressed; HCC, hepatocellular carcinoma.
